# Assessment of Covid-19 vaccine confidence among healthcare personnel in the safety-net sector in the United States and Puerto Rico

**DOI:** 10.1186/s12913-024-10996-z

**Published:** 2024-05-03

**Authors:** Thomas T. Miles, Shang-Ju Li, Tija Danzig, Miguel Marrero, Ivelisse Morales, Saleh Babazadeh

**Affiliations:** 1 Americares, Stamford, CT USA; 2 Americares, San Juan, PR USA

**Keywords:** COVID-19, Healthcare worker, Safety-net sector

## Abstract

**Background:**

This study aimed to assess COVID-19 vaccine confidence among healthcare personnel in the safety net sector of the United States and Puerto Rico. This study aimed to examine the extent to which increased knowledge and positive attitudes toward COVID-19 vaccine safety and efficacy were associated with healthcare workers’ COVID-19 vaccination status and their recommendation of the vaccine to all patients.

**Methods:**

Online survey data were collected from health care workers working in Free and Charitable Clinics across the United States and Federally Qualified Health Centers in Puerto Rico. The survey consisted of 62 questions covering various demographic measures and constructs related to healthcare workers’ vaccination status, beliefs, and recommendations for COVID-19 vaccination. Statistical analyses, including multivariate analysis, were conducted to identify the factors associated with the COVID-19 vaccine status and recommendations among healthcare personnel.

**Results:**

Among the 2273 respondents, 93% reported being vaccinated against COVID-19. The analysis revealed that respondents who believed that COVID-19 vaccines were efficacious and safe were three times more likely to be vaccinated and twice as likely to recommend them to all their patients. Respondents who believed they had received adequate information about COVID-19 vaccination were 10 times more likely to be vaccinated and four times more likely to recommend it to all their patients.

**Conclusions:**

The study results indicate that healthcare workers’ confidence in COVID-19 vaccines is closely tied to their level of knowledge, positive beliefs, and attitudes about vaccine safety and efficacy. The study emphasizes the significance of healthcare workers feeling well informed and confident in their knowledge to recommend the vaccine to their patients. These findings have important implications for the development of strategies to boost COVID-19 vaccine confidence among healthcare workers and increase vaccine uptake among patients.

**Supplementary Information:**

The online version contains supplementary material available at 10.1186/s12913-024-10996-z.

## Background

In 2019, a new virus, coronavirus disease 2019 (COVID-19), first surfaced in China and rapidly spread all over the world, creating a pandemic. By March 2022, more than 472 million COVID-19 cases were confirmed and more than six million COVID-19 confirmed deaths worldwide [1]. An acute respiratory disease with high transmissibility requires many healthcare workers to serve patient-facing roles and be exposed to the virus. The prevalence of COVID-19 among health care workers varies, with studies reporting rates of 7% [2], 10.1% [3], and 19% [4]. The highest prevalence was found among nurses and midwives [5]. Despite its high prevalence, the severity and mortality rates among infected healthcare workers are lower than those in the general population [3, 6]. The risk of infection is higher in patient-facing roles [7].

In December 2020, the FDA authorized two vaccines for COVID-19, namely Pfizer-BioNTech COVID-19 Vaccine and Moderna COVID-19 Vaccine. In February 2021, Johnson and Johnson approved a COVID-19 vaccine [8]. The COVID-19 vaccine rollout offered hope for a return to normalcy, but discussions about vaccine hesitancy arose as program success relies on population uptake [9–12]. The success of any immunization campaign relies on both the effectiveness and individuals’ acceptance of the vaccine to reach that goal; thus, the reasons underlying reduced vaccine confidence, particularly among vulnerable groups, must be investigated and addressed as soon as possible.

Understanding COVID-19 vaccine hesitancy among healthcare workers, particularly those in the FCC and FQHC in Puerto Rico, is crucial for several reasons. First, healthcare workers are at the forefront of the pandemic response and their vaccination status can influence public trust and vaccine uptake [13]. Second, vaccine hesitancy among this group is influenced by concerns about safety and efficacy as well as mistrust of the government and institutions [14]. Third, vaccine hesitancy is prevalent among healthcare workers, with concerns about safety, efficacy, and potential side effects being the top reasons [15]. Finally, educational interventions targeting safety concerns, vaccine efficacy, and sense of duty are crucial in addressing vaccine hesitancy among healthcare workers [16]. Therefore, understanding and addressing vaccine hesitancy among healthcare workers in the FCC and FQHC in Puerto Rico is essential for improving vaccine uptake and controlling the spread of COVID-19.

Vaccine Hesitancy is defined as a delay in acceptance or refusal of vaccination despite the availability of vaccination services. Vaccine hesitancy is complex and context-specific, and varies across time, place, and vaccines [17]. The World Health Organization (WHO) identified vaccine hesitancy as one of the top ten threats to global health in 2019 [18]. Many studies and reports on vaccine hesitation from the United States, China, the United Kingdom, Ireland, and Congo have shown that vaccination acceptance and hesitancy of the general population and healthcare workers vary in many settings [19–22].

.However, recent studies in the US and worldwide have revealed an acceptance rate between 31% and 86% and a rapidly changing environment [23–25]. COVID-19 vaccination hesitancy among healthcare workers is a complex issue influenced by various factors such as perceived risk, fear of infection, and beliefs about the vaccine. Understanding these factors is crucial for developing targeted interventions to increase vaccine acceptance among healthcare workers. COVID-19 vaccination hesitancy among healthcare workers has been the subject of significant research interest. Studies have reported varying levels of vaccine acceptance among healthcare workers, with some studies indicating low acceptance rates. For instance, a study in Egypt found that only 28% of healthcare workers accepted the COVID-19 vaccination [26]. Similarly, a pilot study in the US reported surprisingly high levels of vaccine hesitancy among healthcare workers, with 23% of correctional healthcare workers and 17% of general healthcare workers refusing to be vaccinated against COVID-19 [27]. Moreover, a study in Guinea used logistic regression to identify vaccination-associated factors for COVID-19 among healthcare workers and the general population [28]. The study found that both facilitators and barriers to COVID-19 vaccination exist among healthcare workers. Several factors have been associated with hesitancy to receive the COVID-19 vaccine among healthcare workers. A systematic review exploring COVID-19 vaccine hesitancy among healthcare workers found that direct contact/care with COVID-19 patients or higher perceived risk and fear of being infected with COVID-19 was associated with lower COVID-19 vaccination hesitancy in more than half of the studies [29]. Additionally, a study in India found that beliefs about the vaccine were not uniform among healthcare workers, leading to hesitancy and negatively affecting the general population’s perception of COVID-19 vaccination [30]. Furthermore, a study in Switzerland assessed opinions on COVID-19 vaccination, willingness to be vaccinated, and reasons for vaccination hesitancy among healthcare workers, highlighting the multifaceted nature of vaccine hesitancy [31]. The rapid evolution of the COVID-19 pandemic has made it difficult to gather empirical evidence on the factors that contribute to vaccine hesitancy among healthcare workers. This study aimed to investigate the factors affecting COVID-19 vaccine hesitancy and acceptance among healthcare workers employed in Free and Charitable Clinics (FCC) and Federally Qualified Health Centers (FQHC) in the United States and Puerto Rico. Despite the scarcity of empirical data, COVID-19 vaccine hesitancy among healthcare workers remains largely unexplored. Americares extend their support to a network of free and charitable clinics and federally qualified health centers spread across the United States and Puerto Rico. These clinics cater to vulnerable individuals who, without such facilities, would have no access to healthcare, namely, those who are uninsured or underinsured. The American partner network of FCCs and FQHCs serves a significant population of over seven million patients [17]. Despite their critical role in providing healthcare services to a highly vulnerable segment of the US population, there is a noticeable lack of research on this sector in the literature.

In this study, we aimed to understand COVID-19 vaccine hesitancy among healthcare workers, particularly those in the FCC and FQHC in Puerto Rico for several reasons. First, healthcare workers are at the forefront of the pandemic response, and their vaccination status can influence public trust and vaccine uptake [13]. Second, vaccine hesitancy among this group is influenced by concerns about safety and efficacy, as well as mistrust of government and institutions [14]. Third, vaccine hesitancy is prevalent among healthcare workers, with concerns about safety, efficacy, and potential side effects being the top reasons [15]. Finally, educational interventions targeting safety concerns, vaccine efficacy, and a sense of duty are crucial in addressing vaccine hesitancy among healthcare workers [16]. Therefore, understanding and addressing vaccine hesitancy among healthcare workers in FCC and FQHC in Puerto Rico is essential for improving vaccine uptake and controlling the spread of COVID-19.

## Methods

### Study setting and population

Without a universal health insurance scheme, The United States, with its large under-or uninsured population, has developed a healthcare safety net, or a sector of the healthcare system largely catering to the healthcare needs of those with no or limited insurance, as well as many Medicaid beneficiaries. Historically, the safety net sector consists of federally qualified health centers (FQHC). Clinics that receive funding through the U.S Health Resources and Service Administration (HRSA) to offer primary care services to underserved populations and free and charitable clinics (FCC), private not for profit facilities, constitute a patchwork of institutions, clinics, independent physicians, and other providers supported by a plethora of funding strategies that vary based on local political and economic environments as well as the number of local under or uninsured individuals [32]. Healthcare safety net personnel are those individuals who work at facilities that deliver a significant level of healthcare to under-or uninsured individuals and Medicaid recipients and, either by legal mandate or explicit adoption of a mission, offer care to patients regardless of their ability to pay for those services [33]. A 2010 census estimated approximately 1400 FCC within the 50 US states, while in Puerto Rico 21, FQHC serves a similar population [34].

### Study design

This project was part of a larger effort to evaluate the impact of CDC’s Vaccinate with the Confidence Program. *Vaccination with Confidence* is CDC’s strategic framework to strengthen vaccine confidence and prevent outbreaks of vaccine-preventable diseases in the United States. A cross-sectional online survey was conducted among healthcare personnel in FCC and FQHC across mainland United States and Puerto Rico between June and August 2021. Healthcare personnel (HCP) include both clinical (Physician, Nurse practitioner, physician assistant nurse, nurse assistant, and diagnostic technician) and non-clinical staff (administrative personnel, custodial, and food service) to align with the guidance from the US Center for Disease Control and Prevention, Division of Healthcare Quality Promotion. Using non-probability convenience sampling, invitations to complete the survey were sent to a sample of FCCs and FQHCs. The access link to the survey was sent to the directors of clinics who were members of either the National Association of Free and Charitable Clinics (NAFC), State Associations of free and charitable clinics, or FQHCs in Puerto Rico. Clinic Directors were then asked to share the survey link with their staff. HCP who was at least 18 years of age and gave written consent were asked to participate. Those who did not sign the consent form were excluded from analysis. The survey was conducted by both clinical and non-clinical healthcare staff members. Including non-clinical staff from free and charitable clinics in studies on COVID-19 vaccine recommendations can be particularly crucial because of the unique position of these facilities in the healthcare system. Free and charitable clinics often serve as primary healthcare resources for underserved and vulnerable populations, making the attitudes and behaviors of all staff members towards vaccination vitally important. Non-clinical staff in these settings, even without direct patient care roles, can significantly impact patients’ perceptions and decisions regarding vaccines due to their close interaction with the community and their role in creating an environment that prioritizes health and safety. Their inclusion in vaccine recommendation studies underscores their commitment to comprehensive public health strategies across all healthcare settings, ensuring that the measures taken to encourage vaccination are inclusive and consider the perspectives of those working in all capacities within healthcare, especially in facilities that are most vulnerable.

The survey, consisting of 62 questions, covered various demographic measures and constructs from the Theory of Reasoned Action (TRA) and the Health Belief Model (HBM), as they relate to either the HCP vaccination for COVID-19 or HCP recommendation of COVID-19 vaccination to all patients in their clinic setting [35–37]. These constructs were assessed using five-point Likert scale questions [38]. The choices were I strongly agree,” I do not agree or disagree, I disagree, and I strongly disagree. These constructs included knowledge of the COVID vaccine (3 questions), risk perception of COVID infection (2 questions), attitude towards COVID-19 vaccine (8 questions), and attitude towards their ability to answer patients’ questions regarding COVID-19 vaccine (5 questions). The survey was conducted in both English and Spanish and took approximately 20 min to complete.

### Data management and analysis

The survey was programed for online application utilizing KoBo Toolbox, a data collection, management, and visualization platform used globally for research and social good [39]. Participants responded to the survey after receiving the link. Although the attitude questions designed for this survey used a five-point Likert scale, for the current analysis, the Likert-scale questions were converted to a binary variable showing the expected positive attitude. For this conversion, “I strongly agree” and “I agree” were coded as 1 (positive attitude) and “I do not agree or disagree”, “I disagree”, and “I strongly disagree” were coded as 0 The association of multiple knowledge and attitude variables with two main outcome variables was tested: 1- Whether the HCW is vaccinated against COVID-19 ; and 2-Whether the HCW recommends the COVID-19 vaccine to all their patients. A descriptive analysis of demographic and two-sided t-test and chi-2 test were used to examine associations between each of the demographic and TRA and HBM constructs, and COVID-19 vaccination and COVID-19 vaccination recommendation behaviors. Multivariate logistic regression models were constructed to examine the association between each of the variables and COVID-19 vaccination and vaccination recommendation behaviors. Additional demographic variables, which were found to be related to vaccination in previous studies, were included in the models along with those variables that showed an association with vaccination at a significance level of 0.25. All findings with P-values of < 0.05 were considered “statistically significant.” Analyses were conducted using Stata/SE 15 [40].

This study received ethical approval from the WIRB-Copernicus Group (WCG® IRB) and confidentiality measures were implemented for all participants. The relevant ethical considerations and approval documents, with tracking number 20,213,318, are accessible at: https://www.wcgirb.com/.

## Results

Table 1 presents a summary of the respondents’ individual characteristics. The survey was completed by 2273 healthcare workers across 36 states and Puerto Rico. The COVID-19 vaccination rate for the entire sample was 92.6%. Of the total participants, 83.1% indicated that they would advise all patients on the vaccine. The age distribution of the participants indicated that 25.1% were over 65 years old and only 9.9% were younger than 25 years. The majority of respondents were female (79.2%), white (74.8%), and non-Hispanic (76.7%). Notably, more than half of the respondents (55.1%) held non-clinical positions and were formally employed (56.9%) by their respective clinics rather than working in volunteer positions.

**Table 1:** Individual characteristics of survey respondents

Variable	N (%)
Age	
< 25	224 (9.9)
25–34	344 (15.1)
35–44	339 (14.9)
45 = 54	402 (17.7)
55–64	393 (17.3)
≥ 65	571 (25.1)
Sex	
Male	461(20.3)
Female	1800 (79.2)
Prefer not to say	12 (0.5)
Race	
White	1,700 (74.8)
Non-white	573 (25.2)
Hispanic	
Yes	529 (23.3)
No	1,744 (76.7)
Position	
Clinical	1,021(44.9)
Non-clinical	1,252 (55.1)
Employment	
Employed	1,294 (56.9)
Volunteer	979 (43.1)
COVID-19 Vaccination	
Yes	2105 (92.6)
No	168 (7.4)
Recommend to all patients	
Yes	1888 (83.1)
No	384 (16.9)
Total	2273 (100.0)

Table 2 shows the bivariate analysis of the independent variables and the outcome variables of interest. Bivariate analysis showed that age, sex, knowledge of vaccine types, knowledge of vaccine doses, and attitude questions were significantly associated with vaccination status. In addition, age, position, knowledge of effectiveness, knowledge of costs, and all other attitude questions were significantly associated with the recommendation of the vaccine to all patients.

**Table 2:** Bivariate analysis of outcomes and independent variables

	N	HCW is vaccinated for COVID-19		HCW recommends the vaccine to all patients	
		%	p-value*	%	p-value*
Age					
< 25	224	92	< 0.001	80.8	0.023
25–34	344	87.2		81.4	
35–44	339	89.4		81.4	
45–54	402	92.5		85.1	
55–64	393	94.4		79.6	
> 65	571	96.9		87	
Position					
Non-clinical	1252	92.1	0.298	81.7	0.049
Clinical	1021	93.2		84.8	
Race					
White	1700	92.8	0.5	82.2	0.056
Non-white	573	92		85.7	
Sex					
Woman	1800	92.3	0.02	82.8	0.182
Man	461	94.4		84.8	
Hispanic Origin					
Non-Hispanic	1744	92.8	0.582	82.2	0.042
Hispanic	529	92.1		86	
Knowledge of 3 vaccines					
No	104	79.8	< 0.001	78.9	0.235
Yes	2169	93.2		83.3	
Knowledge of doses					
No	151	78.8	< 0.001	82.1	0.738
Yes	2122	93.6		83.2	
Knowledge of effectiveness					
No	285	75.1	< 0.001	67.4	< 0.001
Yes	1988	95.1		85.4	
Knowledge of cost					
No	88	79.6	< 0.001	75	< 0.001
Yes	2185	93.1		83.4	
Believe vaccines decrease risk of infection					
No	197	57.9	< 0.001	52.8	< 0.001
Yes	2076	95.9		86	
FDA vaccines are efficacious and safe					
No	1011	85.2	< 0.001	75.4	< 0.001
Yes	1262	98.6		89.3	
Concerned about side-effects					
No	135	66.7	< 0.001	57.8	< 0.001
Yes	2138	94.3		84.7	
Received adequate info regarding vaccine					
No	212	41	< 0.001	34	< 0.001
Yes	2061	97.9		88.2	
Feel confident to answer all 4 patients’ questions					
No	942	87.7	< 0.001	74.2	< 0.001
Yes	1331	96.1		89.4	
Vaccine is a good idea for patients					
No	203	46.3	< 0.001	36	< 0.001
Yes	2070	97.2		87.7	

* Association was tested using the Chi-square test. *p* < 0.05 considered significant

Table 3 shows the respondents’ knowledge, attitudes, and COVID-19 vaccination practices by race, sex, and Hispanic ethnicity. More than 90% of the respondents believed that vaccination decreased the risk of infection. Only 60% of respondents believed that FDA vaccines are efficacious, and three-quarters of respondents believed that FDA-approved vaccines are safe. Approximately a quarter of the respondents were concerned about the side effects of the COVID vaccine. Our results showed that 94% of respondents believed that they had received adequate information about COVID-19 vaccination. Nine out of 10 respondents believed that receiving a COVID 19 vaccine was a good idea, and around 93% of the respondents had already received a COVID vaccine.

The majority of healthcare workers surveyed expressed confidence in answering patients’ questions regarding COVID-19 vaccine access, with 90.6% feeling confident in this area. However, confidence was lower in answering questions about vaccine development, with only 64.9% of the respondents feeling confident in this area. However, over three-fourths of the respondents felt confident in addressing queries about vaccine side effects, efficacy, and benefits. A small percentage of respondents (approximately 3%) were unsure about where to direct their patients to COVID-19 vaccination. Furthermore, only 9.0% of the healthcare workers had doubts or did not believe that vaccination against COVID-19 was a good idea for their patients. In general, 83.1% of the healthcare workers indicated that they would recommend COVID-19 vaccination to all their patients.

**Table 3:** Respondents’ knowledge, attitude, and practice toward COVID-19 vaccination

		Gender (%)		Hispanic (%)		Race (%)	
Question	All	Female	Male	Hispanic	Hispanic	White	Non-white
				(N)	(Y)		
Believe vaccine decrease risk of infection	91.3	91.2	92.2	92.8*	86.4*	92.7*	87.3*
FDA vaccines are efficacious	63.0	60.3*	73.8*	70.4*	38.6*	68.6*	46.3*
FDA vaccines are safe	60.6	57.6*	72.7*	67.4*	38.2*	65.8*	45.0*
Concerned about side-effects	26.2	27.7*	19.7*	21.0*	43.3*	22.0*	38.7*
Received adequate info	94.1	94.3	93.3	94.9*	91.3*	95.5*	89.9*
Getting vaccinated is good idea	90.7	90.2*	93.1*	91.1	89.2	91.4	88.7
Got vaccinated	92.6	92.3*	94.4*	92.8	92.1	92.8	92.0
Feel confident to answer access questions	90.6	91.4*	87.4*	91.1	89.0	91.1	89.0
Feel confident to answer vaccine development questions	64.9	63.6*	69.6*	65.3	63.3	65.4	63.4
Feel confident to answer side effect questions	79.0	79.7	76.6	80.3*	74.9*	79.8	76.6
Feel confident to answer efficacy questions	79.3	78.4	82.9	80.5*	75.4*	80.6	75.4
Feel confident to answer benefit questions	87.1	86.6	89.2	88.4*	83.0*	88.2*	83.9*
Know where to refer patients	97.5	98.2*	95.4*	97.7	97.2	98.1*	96.0*
Vaccine is a good idea for patients	91.1	90.9*	92.2*	91.1	90.9	91.4	90.1
Recommend to all Patients	83.1	82.8	84.8	82.2*	86.0*	82.2	85.7

* Association was tested using the chi-squared test. Statistical significance was set at *p* < 0.05. Positive answers were tested against negative answers for all the variables in the table

Tables 4 and 5 show the bivariate and multivariate analyses of the independent variables in relation to the outcome variables of interest. The Adjusted Odds Ratios (OR) illustrate the odds of a healthcare worker recommending the vaccine to a patient based on a specific characteristic or factor relative to the reference group (identified as “Ref.“). If the reference group is not mentioned in the table, then the variable is used as a binary variable. The Confidence Intervals (CI) delineate the range of values within which the true odds ratio is likely to fall.

Table 4 presents a multivariate analysis of the outcomes of COVID-19 vaccination among the surveyed healthcare workers. The outcome was determined by self-report of the question, “Are you vaccinated against COVID-19?” The analysis included questions on age, position in the clinic, race, sex, Hispanic origin, and seven knowledge and belief questions. After accounting for these variables, the results showed that healthcare workers over 65 were 2.8 times more likely to be vaccinated than the reference group. Additionally, healthcare workers who believed that vaccination decreased the risk of infection were nearly twice as likely to be vaccinated. Respondents who believed that FDA-approved COVID-19 vaccines were effective and safe were three times more likely to be vaccinated. Healthcare workers who reported receiving adequate information about COVID vaccines were approximately 10 times more likely to be vaccinated against COVID-19. The results also showed that healthcare workers who believed that vaccination was a good idea for all patients were nearly four times more likely to be vaccinated. Finally, respondents who indicated that they would recommend the vaccine to all their patients were 2.4 times more likely to be vaccinated.

**Table 4:** Multivariate analysis of whether HCW is vaccinated against COVID-19

	HCW is vaccinated for COVID-19
	Adjusted OR (95% CI)
Age	
< 25	Ref.
25–34	0.7 (0.3–1.5)
35–44	0.9 (0.4–2)
45–54	1.3 (0.5–2.9)
55–64	1.9 (0.8–4.7)
> 65	2.8** (1.1–7.4)
Position (Clinical)	1.0 (0.6–1.6)
Race (none-white)	1.5 (0.9–2.6)
Sex (Female)	1.0 (1.0–1.0)
Hispanic	1.6 (0.9–2.8)
Knowledge of 3 vaccines	2.3** (1.0–5.0)
Knowledge of doses	2.0** (1.0–4.0)
Knowledge of effectiveness	1.6 *(1.0–2.8)
Knowledge of cost	1.1 (0.5–2.4)
Believe vaccines decrease the risk of infection	1.5 (0.9–2.7)
FDA vaccines are efficacious and safe	2.7*** (1.5–5.0)
Concerned about side-effects	0.7 (0.4–1.3)
Received adequate info	10.1*** (5.4–18.8)
Feel confident to answer all 4 questions	0.9 (0.5–1.5)
The vaccine is a good idea for patients	3.6*** (1.9–6.6)
Recommend vaccine to all Patients	2.6*** (1.6–4.4)

*p < 0.1 **p < 0.05 ***p < 0.01, CI: confidence interval, OR: odds ratio, HCW: health care worker

Table 5 presents a multivariate analysis of the recommendations for COVID-19 vaccination for all patients. The outcome was determined by the question, “Would you recommend the COVID-19 vaccine to all your patients?” and was controlled for age, position in the clinic, race, sex, Hispanic origin, vaccination status, and six knowledge- and attitude-related questions. The results indicate that healthcare workers in the 45–54 age group and over 65 years are 60% more likely to recommend the vaccine to all their patients. Additionally, those of Hispanic and non-white origin had 50% and 60% higher odds of recommending the vaccine to all their patients, respectively.

Our analysis also revealed that respondents who were already vaccinated were 2.6 times more likely to recommend the vaccine to their patients. Furthermore, healthcare workers who believed that FDA-approved vaccines were efficacious and safe were 1.5 times more likely to recommend vaccinating their patients against the virus. Among all respondents, those who thought they had received adequate information about the vaccine were almost four times more likely to recommend vaccination to all their patients. The odds of recommending the vaccine to all patients were nearly two times higher among respondents who felt confident answering all four types of questions about the COVID vaccine. Finally, those who believed that vaccination was a good idea for patients had 2.7 times higher odds of recommending the COVID-19 vaccine to all patients.

**Table 5:** Multivariate analysis of whether HCW recommend the vaccine to all patients

	HCW recommends the vaccine to all patients
	Adjusted OR (95% CI)
Age	
< 25	Ref.
25–34	1.2 (0.7–2)
35–44	1.3 (0.8–2.1)
45–54	1.6* (1–2.7)
55–64	1 (0.6–1.6)
> 65	1.6* (1–2.6)
Position (Clinical)	1.0 (0.7–1.3)
Race (none-white)	1.6*** (1.2–2.3)
Sex (Female)	1.0 (1.0–1.0)
Hispanic	1.5** (1.1–2.1)
Knowledge of 3 vaccines	1.0 (0.5–1.9)
knowledge of doses	0.4 (0.2–0.8)
Knowledge of effectiveness	1.1 (0.7–1.6)
Knowledge of cost	0.7 (0.4–1.4)
Believe vaccines decrease the risk of infection	1.2 (0.8–2)
FDA vaccines are efficacious and safe	1.5*** (1.2–2)
Concerned about side-effects	0.9 (0.5–1.5)
Received adequate info	3.6*** (2.2–6)
Feel confident to answer all 4 questions	1.9***(1.4–2.5)
The vaccine is a good idea for patients	2.8*** (1.7–4.4)
HCW got vaccinated	2.6*** (1.6–4.3)

*p < 0.1 **p < 0.05 ***p < 0.01, CI: confidence interval, OR: odds ratio, HCW: health care worker

## Discussion

The results of the study indicate that the vaccination rates among personnel at safety net health facilities as of June 2021 were high at approximately 92.6%. Additionally, 83.1% of respondents indicated that they would recommend the vaccine to all patients. The findings also suggest that healthcare workers’ knowledge of COVID-19 vaccines plays a critical role in getting vaccinated and recommending the vaccine to patients. Moreover, the study suggests that perceived knowledge about the vaccine was a significant factor in getting vaccinated and recommending it to all the patients. This aligns with the research by, which highlighted variances in COVID-19 vaccine acceptance rates among healthcare workers across different regions, emphasizing the influence of factors such as knowledge and intention to accept the vaccine [41]. Additionally, this study resonates with the work of, which reported varying rates of vaccine acceptance among healthcare professionals, emphasizing the impact of state recommendations on vaccination decisions [42]. Furthermore, the study’s emphasis on the role of knowledge in vaccination decisions is supported by a study that explored the acceptability of COVID-19 vaccination among healthcare workers and highlighted the importance of understanding the factors influencing vaccine acceptance [43]. Our findings, however, found no effect of clinical versus non-clinical position on vaccination, which contradicts other studies that identified clinical staff as more likely to agree to COVID-19 vaccination compared to non-clinical staff members, emphasizing the significance of healthcare professionals’ attitudes towards vaccination [44].

The study also revealed that individuals of Hispanic or non-white ethnicity were less likely to believe that COVID-19 vaccines can decrease the risk of infection, perceive lower efficacy and safety of the vaccines, express greater concern about side effects, and feel less informed about COVID-19 vaccines. In contrast, identifying as female was associated with lower perceptions of vaccine efficacy and safety, heightened concerns about side effects, and less favorable attitudes towards vaccination. Despite these associations among race, ethnicity, and sex align with existing research on COVID-19 vaccine hesitancy [45], they are concerning given the enormous burden of COVID-19 in Hispanic and minority communities [46–49].

In the multivariate analysis, HCP vaccination was largely associated with increased knowledge and several positive beliefs and attitudes about COVID-19 vaccine safety and efficacy. In fact, this finding adds to the large body of literature that has been produced since the start of the SARS-COV-2 pandemic examining covariates of vaccination uptake and intention among both the general public and healthcare workers [50–53].

In terms of recommendation behavior, our analysis highlights the role of both HCP attitudes towards COVID-19 vaccines and HCP confidence in their vaccine knowledge. Not only do holding positive beliefs and attitudes concerning vaccine efficacy and safety play a significant role in healthcare personnel decisions to recommend COVID-19 vaccination to their patients, but also their perception of, and confidence in their own sense of being informed and their ability to answer key patient questions. This suggests that if healthcare personnel do not feel confident, they have all the information necessary to address patient concerns and questions they are unlikely to broach the topic with a patient, and much less recommend a course of treatment. This is in line with much of the literature that emphasizes the role of provider recommendations in bolstering vaccine confidence among patients.

Our study found that healthcare personnel who believed they had received sufficient information about COVID-19 vaccines were more likely to get vaccinated themselves and recommend vaccines to their patients. This highlights the importance of adequate education and information for healthcare personnel in order for them to make informed decisions about vaccination and to confidently advocate it to their patients. In fact, the study found that healthcare personnel who had received the COVID-19 vaccine themselves were almost four times more likely to recommend it to all their patients, underscoring the powerful role that personal experience can play in shaping attitudes and behaviors. Interestingly, the positive association between healthcare personnel’s vaccine knowledge, uptake, and advocacy behavior is not unique to COVID-19 vaccines. Previous studies have shown similar patterns with other vaccines, such as the Human Papillomavirus (HPV) vaccine, respiratory syncytial virus (RSV) vaccine, influenza vaccine, Guillain Barr virus vaccine, pertussis vaccine, and meningitis vaccine. These findings suggest that healthcare personnel who are knowledgeable about vaccines and who have positive attitudes towards vaccination are more likely to not only get vaccinated themselves but also to advocate for vaccination among their patients. This highlights the important role that health care personnel can play in promoting vaccine uptake and improving public health outcomes [54].

This study adds to the existing research on healthcare personnel behavior during the SARS-CoV-2 pandemic and vaccine hesitancy towards COVID-19 by analyzing a substantial sample of individuals from free and charitable clinics, as well as federally qualified health centers across the United States and Puerto Rico. However, this study has some limitations, one of which is the use of convenience sampling. This may have introduced bias and resulted in low response rates among those who had not received the COVID-19 vaccine at the time of data collection. In addition, distributing a survey link through clinic directors to their healthcare workers might introduce bias by limiting responses to those workers who are more engaged or favored by the directors, potentially skewing the results towards a non-representative subset of the workforce. Additionally, this method may overlook diverse perspectives within the clinic, as workers who are less accessible to directors or less inclined to participate in surveys distributed in such a manner may not have their voices heard, affecting the accuracy and generalizability of the survey outcomes. Another limitation is the nature of the self-report used in this study. The vaccination rate may not be accurate and cannot be generalized to all clinics in the safety net network. Although attempts were made to encourage individuals who were hesitant to receive the vaccine to participate in the study, at the time of data collection, the COVID-19 pandemic and vaccination had become highly politicized, which may have contributed to bias and affected the results [55–57] and likely increased the reticence to participate in many hesitant individuals may have felt [58].

The role that knowledge and understanding of the vaccine play in the decision of healthcare workers to receive vaccination is of great importance, according to research. However, other studies indicate that modifying the attitudes of healthcare workers may require further efforts. The study by Khubchandani et al. (2022) highlights that 20.7% of nurses worldwide refused to be vaccinated against COVID-19. This refusal among nurses could be attributed to various factors such as concerns about vaccine safety, side effects, fear of contracting COVID-19 despite vaccination, and doubts about vaccine efficacy [59]. Additionally, the study by Gu et al. (2022) indicates that healthcare workers, including nurses, exhibit vaccine hesitancy, with 23% of correctional healthcare workers and 17% of general healthcare workers refusing to be vaccinated against COVID-19. This hesitancy may stem from factors like lack of trust in the vaccine, misinformation, and personal beliefs [27].

Finally, as the pandemic has progressed, the definition of vaccination has changed and modified. At the time of data collection, two doses of either the Pfizer or Moderna mRNA vaccine or a single dose of the Johnson & Johnson vaccine were administered. While this study offers key insights into the vaccine-hesitant beliefs of HCP within the safety net sector, since data collection, the addition of several additional doses or boosters has likely changed the vaccination status of much of the sample and extrapolated the findings of this study beyond the initial introduction of COVID-19 vaccines. Although this represents a significant limitation in terms of understanding COVID-19 vaccination rates, it offers valuable insights into understanding and quantifying vaccination coverage during future pandemics.

## Conclusion

In conclusion, the high vaccination rates and willingness to recommend the COVID-19 vaccine among healthcare workers at safety net health facilities underscore the critical role of knowledge and perceived understanding of the vaccine in shaping vaccination behaviors and recommendations to patients. The results highlight the significance of healthcare workers’ perception of the vaccine’s safety and efficacy, as well as their confidence in their knowledge about the vaccine, in influencing their decision to be vaccinated and recommend it to their patients.

### Electronic supplementary material

Below is the link to the electronic supplementary material.

Supplementary Material 1

## Data Availability

The unidentified version of the data used for this study can be shared upon request from the corresponding author ( Saleh Babazadeh).
