# Supplementary material for: Assessment of Covid-19 vaccine confidence among healthcare personnel in the safety-net sector in the United States and Puerto Rico
Source: BMC Health Serv Res. 2024 May 3;24:580. doi: 10.1186/s12913-024-10996-z (PMC11069172; doi:10.1186/s12913-024-10996-z)
Supplement: Supplementary file 1 — Supplementary Material 1 [file 12913_2024_10996_MOESM1_ESM.docx]

Appendix 1:

Survey Instrument:

**CDC Reinforcing Confidence in COVID-19 Vaccines**

**Introduction**

Americares Foundation, a proud and longstanding supporter of free and charitable clinics has partnered with several Free and Charitable Clinic (FCC) State Associations, the National Association of Free and Charitable Clinics, several Federally Qualified Health Centers (FQHC), and the Center for Disease Control (CDC) to understand beliefs, attitudes, and practices around

COVID-19 vaccination among healthcare personnel which includes anyone in a FCC clinical setting ranging from physicians

and nurses, to administrative and custodial personnel. Over the next year, we will be sending out 3 surveys total to understand how attitudes around COVID-19 vaccination are changing.

Please take a moment to complete the following survey which should take no more than **5-10minutes.** Your input is very valuable and will allow us to highlight the FCC and FQHC sector - **shed light on the needs of clinics, providers, other healthcare personnel and the patients they serve. While the first 50 responses will receive a $20 gift card** as compensation for their time, it is our hope that you will find value in your contribution to this effort. We will use the contact information to send the subsequent surveys to you directly but will not share any of your information with anyone outside of our program team.

**Informed Consent**

You are being asked for your consent to take part in a research study regarding healthcare personnel beliefs and attitudes around COVID-19 vaccines. This document provides a concise summary of this research. It describes the key information that we believe most people need to decide whether to take part in this research.

**What should I know about this research?**

- Taking part in this research is voluntary. Whether you take part is up to you.
- If you don't take part, it won't be held against you.
- You can take part now and later drop out, and it won't be held against you.

**How long will I be in this research?** We expect that your taking part in this research will last no more than 10 minutes per survey over the course of 1 year.

**Why is this research being done?**

The purpose of this research is to better understand healthcare personnel beliefs and attitudes around COVID-19 vaccines and how healthcare personnel speak with patients about COVID-19 vaccination.

**What happens to me if I agree to take part in this research?**

If you decide to take part in this research study, the general procedures include taking a series of 3 surveys over the course of the next year.

**Could being in this research hurt me?** This study represents minimal risk. The risks or discomforts you can expect from participating in this research include loss of privacy. For this reason, the clinic you are affiliated with will never have access to your identifiable information. Additionally, survey responses will be stored in a secure and encrypted Americares Foundation online account. Access to this data will be limited to the research team, will be password protected and will be kept for future use. Finally, if participation in the study causes participants to relive an uncomfortable or traumatic experience, participants can leave the survey at any time without repercussions and without affecting their participation in future studies.

**Will being in this research benefit me?**

It is not expected that you will personally benefit from this research.

**Will being in this research benefit others?**

Potential benefits to others include helping public health professionals better understand how to address healthcare personnel concerns regarding COVID-19 vaccination and design strategies so providers can better advise patients on their own concerns.

**If you take part in this study, how will we protect your privacy?**

If you have read this form and have decided to participate in this research, please understand your participation is voluntary and you have the right to withdraw your consent or discontinue participation at any time without penalty or loss of benefits to which you are otherwise entitled. The alternative is not to participate. The results of this research study may be presented at scientific or professional meetings or published in scientific journals. Your individual privacy will be maintained in all published and written data resulting from the study. Results will only be presented using merged responses from all participants and will never reference your individual responses and therefore how you specifically responded to this survey will never be shared.

The information gained here could be used for future research studies or distributed to another investigator for future research studies without additional informed consent from you. Identifiable information acquired as part of this survey will only be used to send you the two subsequent surveys and to provide reminders to participation in this study. You should also know that the WCG Institutional Review Board (IRB), may inspect study records as part of its auditing program, but these reviews will only focus on the researchers and not on your responses or involvement. The IRB is a group of people who review research studies to protect the rights and welfare of research participants.

**Will you be paid/receive credit to take part in the study?** Compensation for being one of the first 50 people nationwide to complete this survey will include a $20 electronic gift card delivered to your email. If you do not complete the study, you will not be eligible for the gift card. Participation is limited to one entry per individual.

**What if you have questions or concerns about the study?** If you have any questions or concerns about the study, whether before or after agreeing to participate, you can call the study Principal Investigator, Dr. Thomas Miles, at 203-658-7418. You can call about any matter having to do with the study, including complaints or questions about your rights as a study participant. This research is being overseen by WCG IRB. An IRB is a group of people who perform independent review of research studies. You may talk to them at 855-818-2289 or [researchquestions@wcgirb.com](mailto:researchquestions@wcgirb.com) if: • You have questions, concerns, or complaints that are not being answered by the research team.• You are not getting answers from the research team.• You cannot reach the research team.• You want to talk to someone else about the research.• You have questions about your rights as a research subject.

**If you agree to participate in this research, please check the box and click next.**

@ Yes, I agree

Q No, I do not agree

**Contact Information**

**Please provide your email address.**

b

**Survey Questions**

**Are you formally employed or a volunteer at the clinic you are affiliated with?**

Q Employed

Q Volunteer

**What is the state/territory of the clinic that you are affiliated with?**

0 Alabama 0 Alaska 0 Arizona

0 Arkansas 0 California 0 Colorado

0 Connecticut 0 Delaware 0 District of Columbia

0 Florida 0 Georgia 0 Hawaii

0 Idaho 0 Illinois 0 Indiana

0 Iowa 0 Kansas 0 Kentucky

0 Louisiana 0 Maine 0 Maryland

0 Massachusetts 0 Michigan 0 Minnesota

0 Mississippi 0 Missouri 0 Montana

0 Nebraska 0 Nevada 0 New Hampshire

0 New Jersey 0 New Mexico 0 New York

0 North Carolina 0 North Dakota 0 Ohio

0 Oklahoma 0 Oregon 0 Pennsylvania

0 Puerto Rico 0 Rhode Island 0 South Carolina

0 South Dakota 0 Tennessee 0 Texas

0 Utah 0 U.S. Virgin Islands 0 Vermont

0 Virginia 0 Washington 0 West Virginia

0 Wisconsin 0 Wyoming

**What is the city of the clinic that you are affiliated with?**


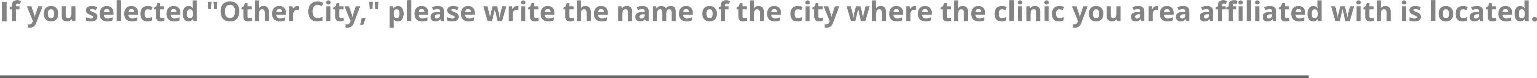


**What is the name of the clinic that you are affiliated with?**


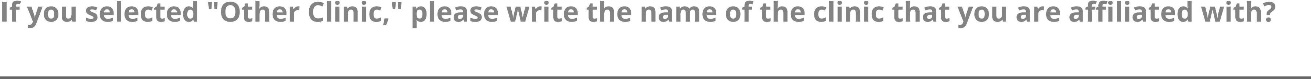


**What is your position at the clinic that you are affiliated with?**

0 Physician

0 Nurse Practitioner

0 Physician Assistant

0 Nurse

0 Nurse Assistant

0 Diagnostic Technician

0 Administrative Personnel

0 Custodial or Food Services

0 Other


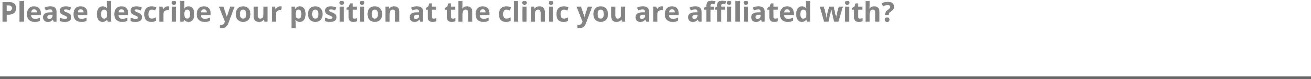


**What is your biological sex?**

0 Man

0 Woman

0 lntersex

0 Prefer not to say

**How do you think of yourself?**

Q Male

Q Female

Q Transgender man/trans man/female-to-male (FTM)

Q Transgender woman/trans woman/male-to-female (MTF)

Q Genderqueer/gender nonconforming neither exclusively male nor female

Q Other

Q Prefer not to answer

**If "other", how would you describe gender identity?**

**Are you of Hispanic/Latino/Spanish origin?**

Q Yes

0 No

**How would you describe your race?** Q American Indian or Alaska Native Q Asian

Q South Asian

Q Black or African American

Q Native Hawaiian or Other Pacific Islander

0 White

Q Other

Q More than one Race

Q Prefer not to say

**How would you describe your race?**


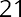

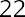

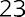

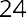

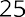

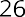

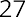

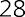

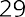

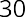

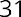

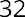

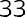

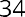

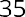

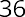

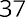

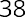

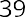

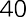

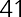

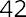

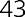

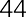

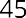

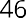

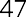

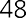

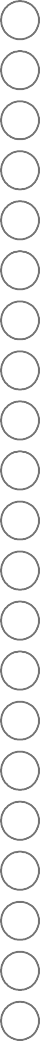

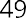

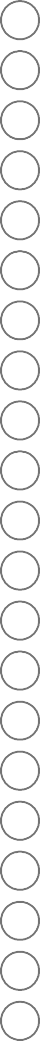

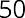

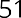

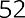

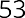

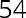

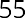

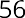

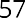

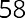

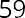

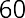

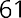

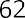

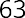

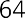

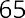

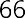

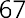

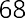

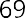

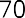

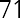

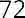

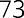

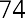

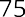

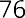

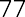

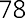

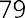

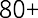


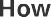

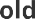

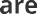

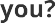


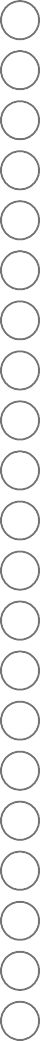

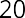


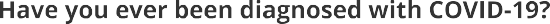

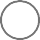

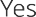

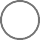

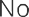

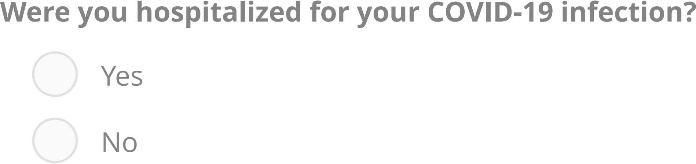

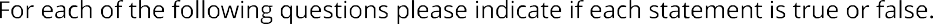


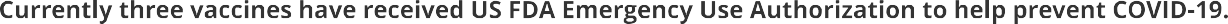

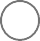

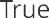

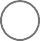

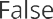

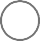


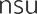

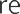


**All US FDA authorized COVID-19 vaccines require 2 doses.**

Q True Q False Q Unsure

**All US FDA authorized COVID-19 vaccines are effective at preventing severe illness or death related to COVID-19.**

Q True Q False Q Unsure

**I know where to access COVID-19 vaccines.**

Q True Q False Q Unsure

**COVID-19 vaccination has no out of pocket costs to the recipient.**

Q True Q False Q Unsure

**I personally know people who have taken a COVID-19 vaccine.**

Q True Q False Q Unsure

**My healthcare provider has recommended that I get a COVID-19 vaccine.**

Q True

Q False

For each of the following questions please indicate your level of agreement with each statement.

**My risk of contracting COVID-19 is high.**


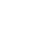

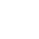

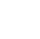

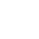

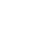


0

0

0

0

0

Strongly Disagree Disagree Neither agree nor

disagree

Agree Strongly Agree

**Contracting COVID-19 would be a serious health concern for me.**


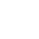

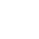

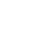

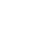

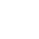


0

0

0

0

0

Strongly Disagree Disagree Neither agree nor

disagree

Agree Strongly Agree

**Vaccination with any of the COVID-19 vaccines decreases the risk of COVID-19 infection.**


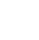

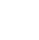

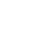

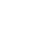


0

0

0

0

0

Strongly Disagree Disagree Neither agree nor

disagree

Agree Strongly agree

**I am concerned about the efficacy of one or more of the US Food and Drug Administration authorized COVID-19 vaccines.**

0

0

0

0

0

Strongly Disagree

Disagree

Neither agree nor disagree

Agree Strongly Agree

**I am concerned about the safety of one or more US Food and Drug Administration authorized COVID-19 vaccines.**

0

0

0

0

0

Strongly Disagree

Disagree

Neither Agree nor Disagree

Agree Strongly Agree

**I am concerned about the side effects of one or more US Food and Drug Administration authorized COVID-19 vaccines.**

0

0

0

0

0

Strongly Disagree

Disagree Neither agree nor disagree

Agree Strongly Agree

**I could access a COVID-19 vaccine easily.**

0

0

0

0

0

Strongly Disagree Disagree

Neither agree nor disagree

Agree Strongly Agree

**I have received adequate information about the COVID-19 vaccines.**

0

0

0

0

0

Strongly Disagree Disagree Neither agree nor

disagree

**Getting a COVID-19 vaccine is a good idea.**

Agree Strongly Agree

0

0

0

0

0

Strongly disagree Disagree Neither agree nor

disagree

Agree Strongly agree

**I have already received a COVID-19 vaccine.**

Q Yes

0 No

The following questions are about your interactions with patients.

**Contracting COVID-19 is a serious health concern for most of my patients.**

| 0 | 0 | 0 | 0 | 0 |
| --- | --- | --- | --- | --- |
| Strongly Disagree | Disagree | Neither agree nor | Agree | Strongly Agree |
|  |  | disagree |  |  |

**Most patients have expressed concern about their risk of contracting COVID-19.**

| 0 | 0 | 0 | 0 | 0 |
| --- | --- | --- | --- | --- |
| Strongly Disagree | Disagree | Neither agree nor | Agree | Strongly Agree |
|  |  | disagree |  |  |

**I feel confident that I could answer patients' questions about how to access a COVID-19 vaccine.**

0

0

0

0

0

Strongly disagree Disagree Neither agree nor

disagree

Agree Strongly agree

**I feel confident that I could answer patients' questions about COVID-19 vaccine development.**

0

0

0

0

0

Strongly disagree Disagree Neither agree nor

disagree

Agree Strongly Agree

**I feel confident that I could answer patients' questions about COVID-19 vaccine side effects.**

0

0

0

0

0

Strongly disagree Disagree Neither agree nor

disagree

Agree Strongly Agree

**I feel confident that I could answer patients' questions about COVID-19 vaccine efficacy.**

0

0

0

0

0

Strongly disagree Disagree Neither agree nor

disagree

Agree Strongly Agree

**I feel confident that I could answer patients' questions about COVID-19 vaccine benefits.**

0

0

0

0

0

Strongly disagree

Disagree

Neither agree nor disagree

Agree Strongly Agree

**Patients getting a COVID-19 vaccine is a good idea.**

0

0

0

0

0

Strongly disagree

Disagree

Neither agree nor disagree

Agree Strongly Agree

**I know where to refer patients to schedule a COVID-19 vaccine.**

Q Yes

0 No

**I personally know of other healthcare professionals who are recommending getting a COVID-19 vaccine to patients.**

Q Yes

0 No

Q Unsure

**I recommend getting a COVID-19 vaccine to ALL patients.**

Q Yes

0 No

**Follow-Up Questions**

**Thank you for completing our survey. If you would like to be reminded about participation in future surveys as part of this project, please provide your phone number.**

As part of this project, we are also planning to conduct multiple interviews and/or focus groups with healthcare personnel from FCC and FQHC about their experience, beliefs, and attitudes around the COVID-19 vaccines. To do this we are seeking expressions of interest in participating. Participants in either an interview or focus group will be compensated for their time and input.

**Would you be interested in participating in either a one-on-one interview or focus group discussion with a member of our study team?**

Q Yes

0 No

**What is your First Name?**

**What is your Last Name?**

**What is the best phone number to reach you?**

Thank you for completing our survey

Thank you for your time.
